# Supplementary figures and images for: Lipopolysaccharide -mediated resistance to host antimicrobial peptides and hemocyte-derived reactive-oxygen species are the major Providencia alcalifaciens virulence factors in Drosophila melanogaster
Source: PLoS Pathog. 2022 Sep 9;18(9):e1010825. doi: 10.1371/journal.ppat.1010825 (PMC9491580; doi:10.1371/journal.ppat.1010825)

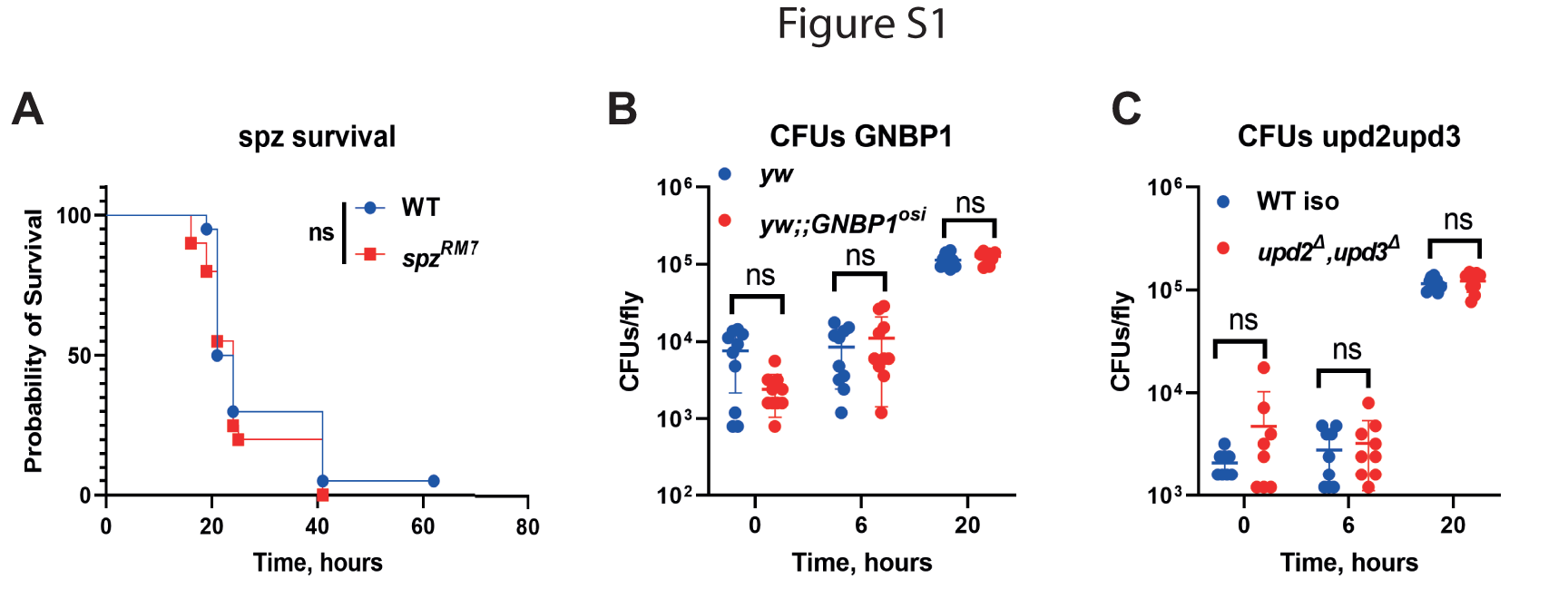

Supplement: S1 Fig — (A) Survival rates of spaetzle mutants and wild-type flies after infection with Pa are similar. (B, C) Pa reaches the same load in GNBP1 (B) and upd2,upd3 (C) mutants as in background control flies. Results (B-C) are shown as mean ± SD of at least 10 samples. Dots represent samples. Each sample included 5 flies and CFUs in each sample were normalized to the number of flies to obtain CFUs per single fly. (TIF) [file ppat.1010825.s001.tif]

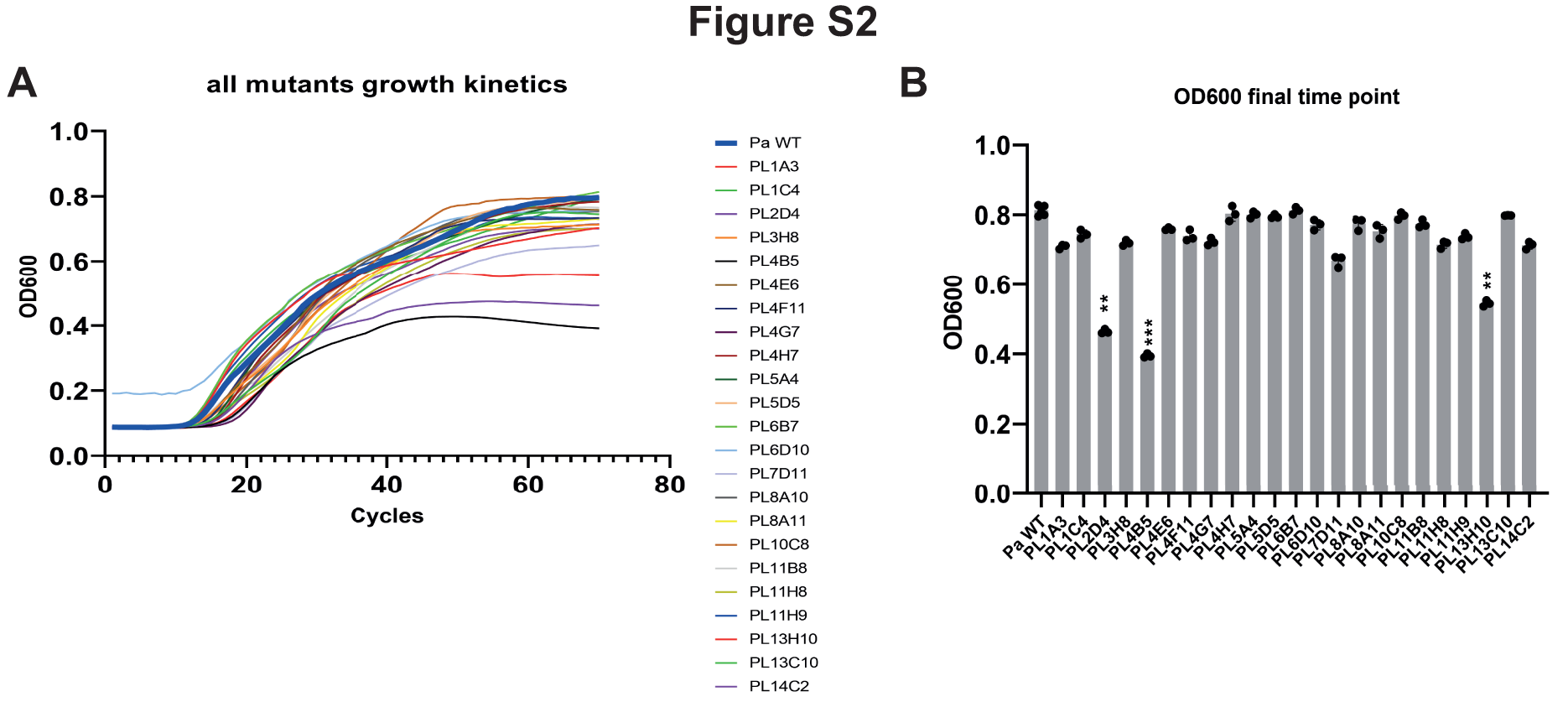

Supplement: S2 Fig — (A, B) Growth kinetics of 23 Pa mutants in LB medium (A) and OD600 at the end of incubation (B) show that only mutants PL2D4, PL4B5, and PL13H10 have significantly lower growth and potentially altered fitness. (TIF) [file ppat.1010825.s002.tif]

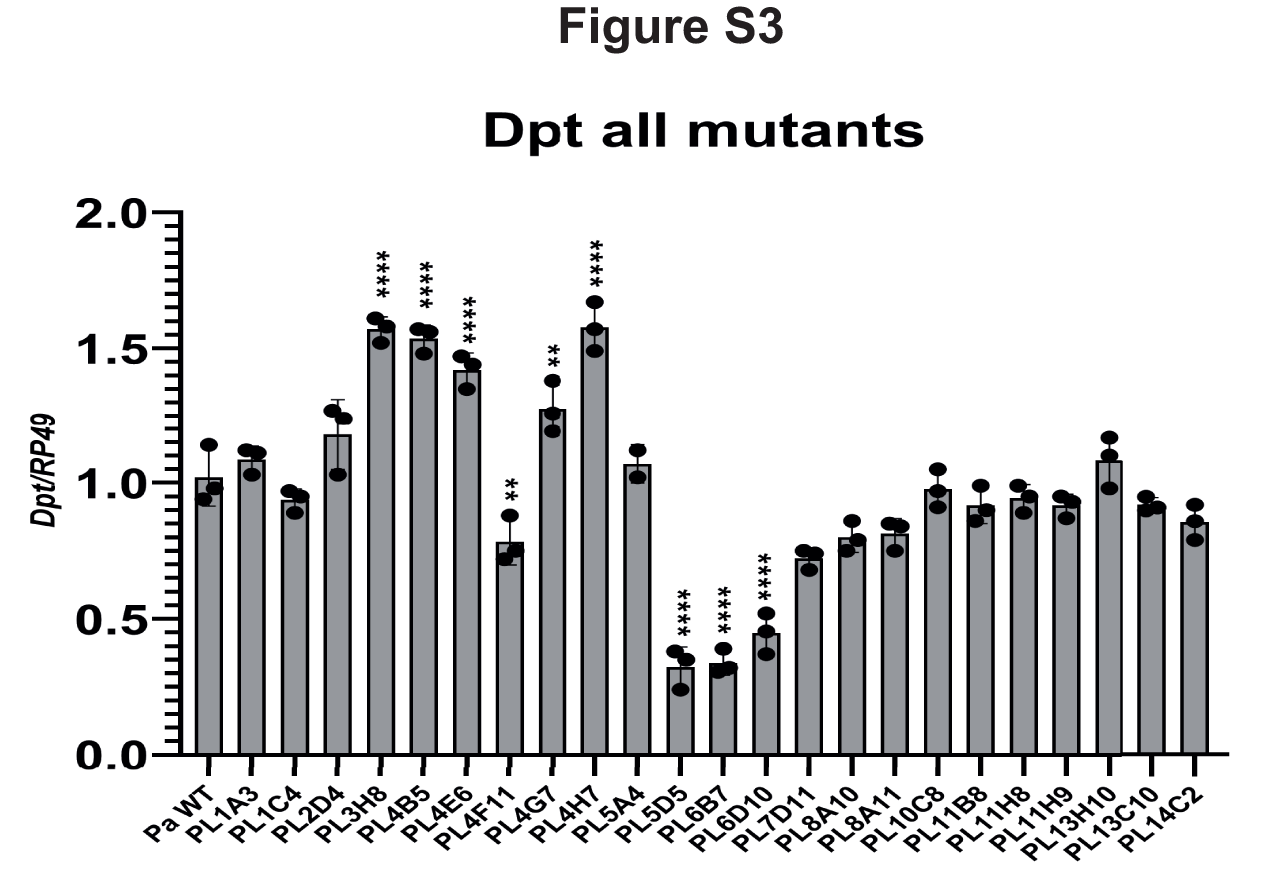

Supplement: S3 Fig — RT-qPCR showing Dpt expression 20 h post infection with attenuated Pa mutants. Asterisks indicate significance relative to Pa WT. (TIF) [file ppat.1010825.s003.tif]

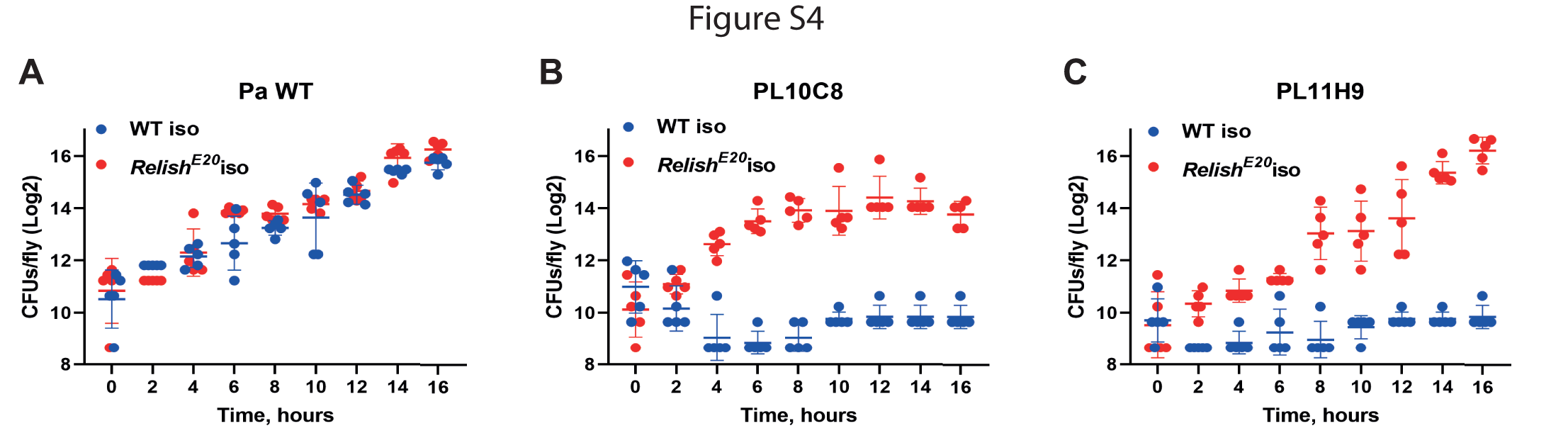

Supplement: S4 Fig — (A-C) Proliferation of wild-type Pa (A), PL10C8 (B), and PL11H9 (C) mutants in wild-type and Relish mutant flies. Wild-type Pa proliferated monotonically in both wild-type and Relish mutant flies (A). The loads of PL10C8 and PL11H9 mutants remained stable in wild-type flies but increased in Relish mutant. Results are shown as mean ± SD of 5 samples (individual flies depicted with dots). (TIF) [file ppat.1010825.s004.tif]

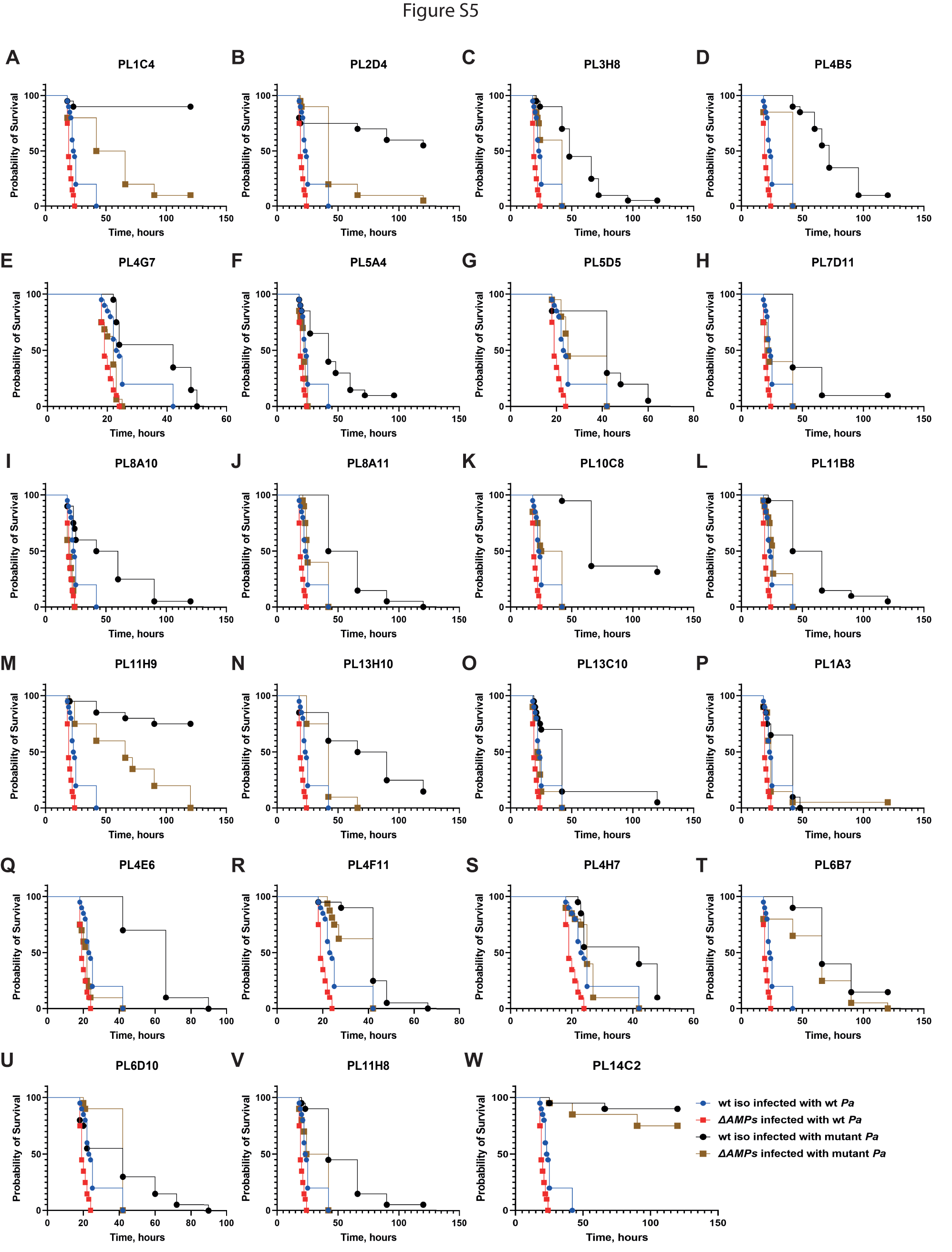

Supplement: S5 Fig — (A-W) Survival rates of 23 attenuated Pa mutants in WT and ΔAMP flies. Vast majority of attenuated mutants retain their virulence in ΔAMP flies. See S3 Table for statistical comparisons. (TIF) [file ppat.1010825.s005.tif]

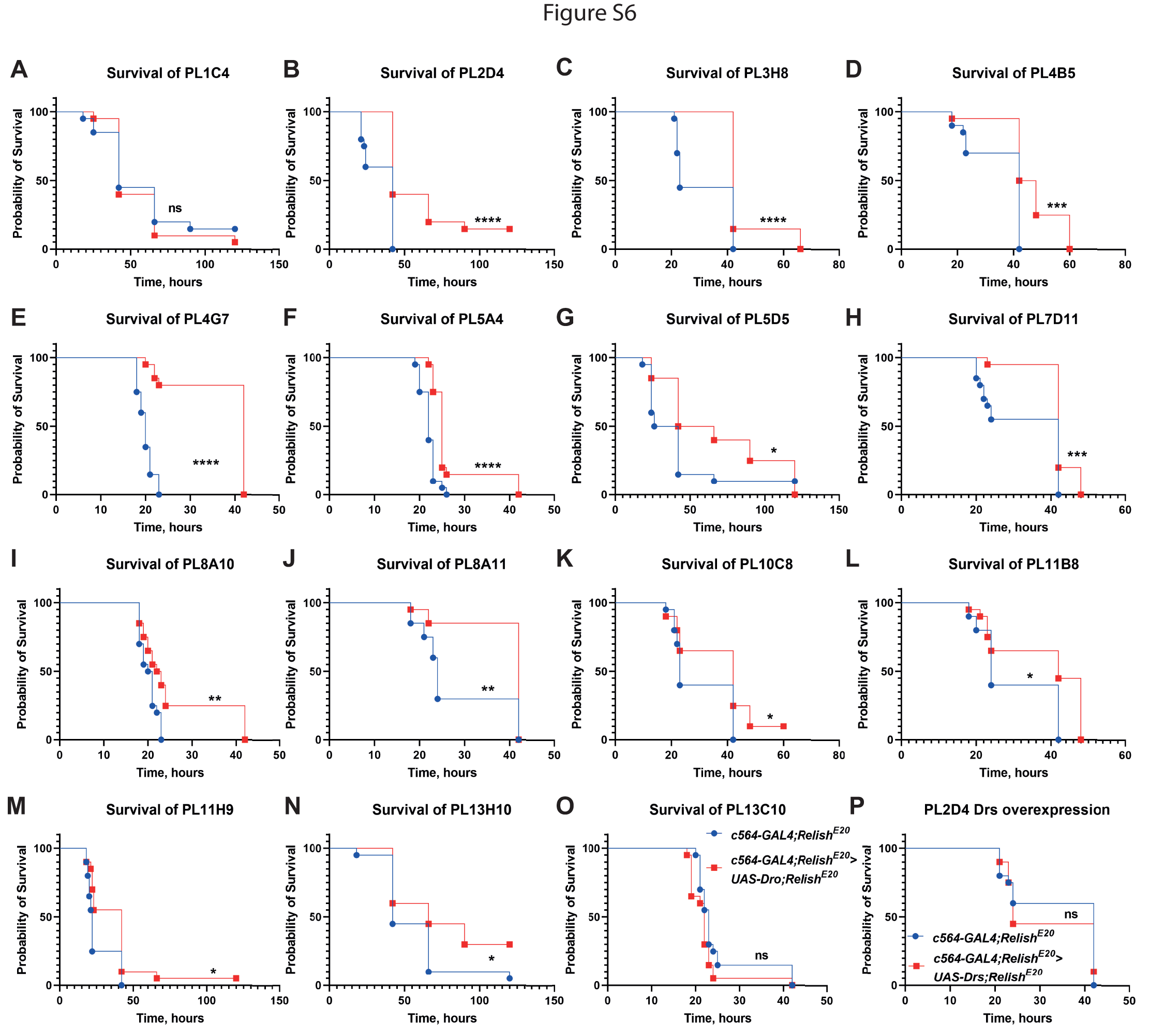

Supplement: S6 Fig — (A-O) Survival rates of Relish control line and Relish line overexpressing Drosocin in fat body after infection with 15 Pa LPS mutants. Only mutants PL1C4 (A) and PL13C10 (O) were not affected by Drosocin overexpression. The other mutants cause significantly lower mortality in overexpression line. (P) Survival rates of Relish control line and Relish line overexpressing Drosomycin in fat body after infection with PL2D4 mutant. No significant improvement of survival was observed in contrast to Drosocin overexpression in panel B. (TIF) [file ppat.1010825.s006.tif]

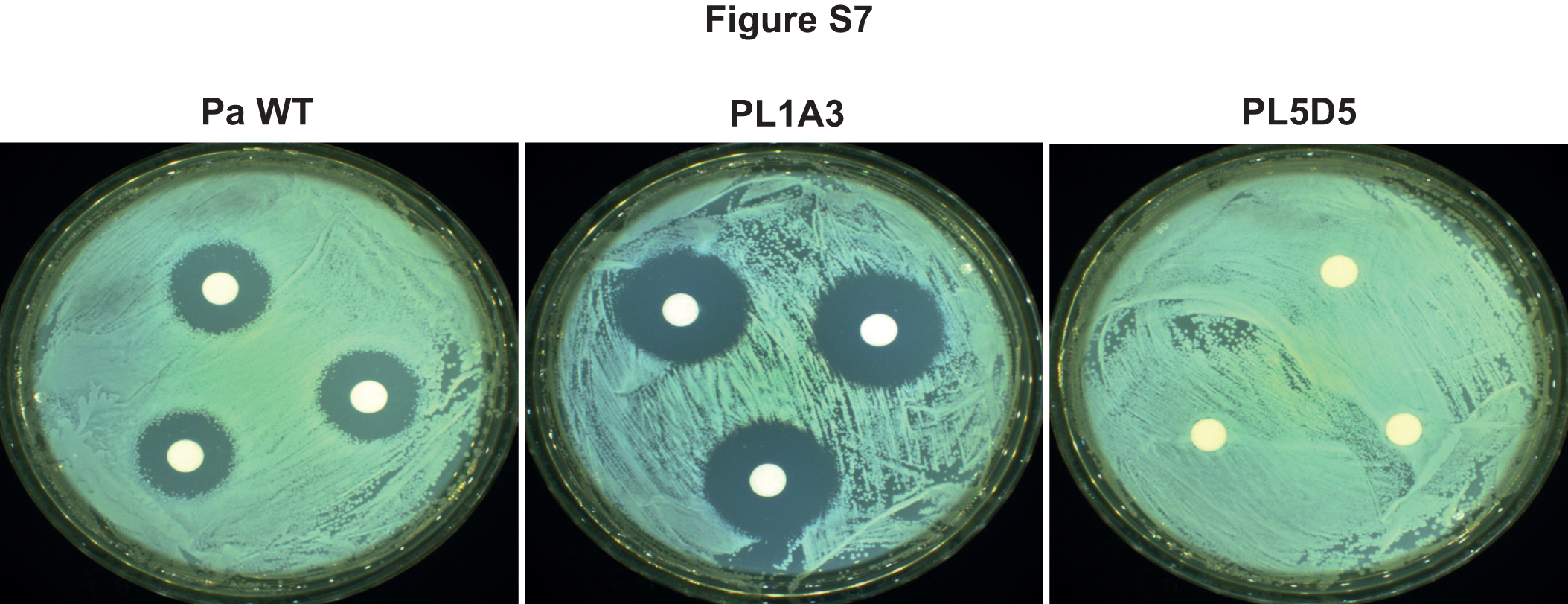

Supplement: S7 Fig — Representative pictures of agar plates showing the size of inhibition zones for WT Pa, ROS-sensitive mutant PL1A3, ROS-resistant mutant PL5D5. (TIF) [file ppat.1010825.s007.tif]

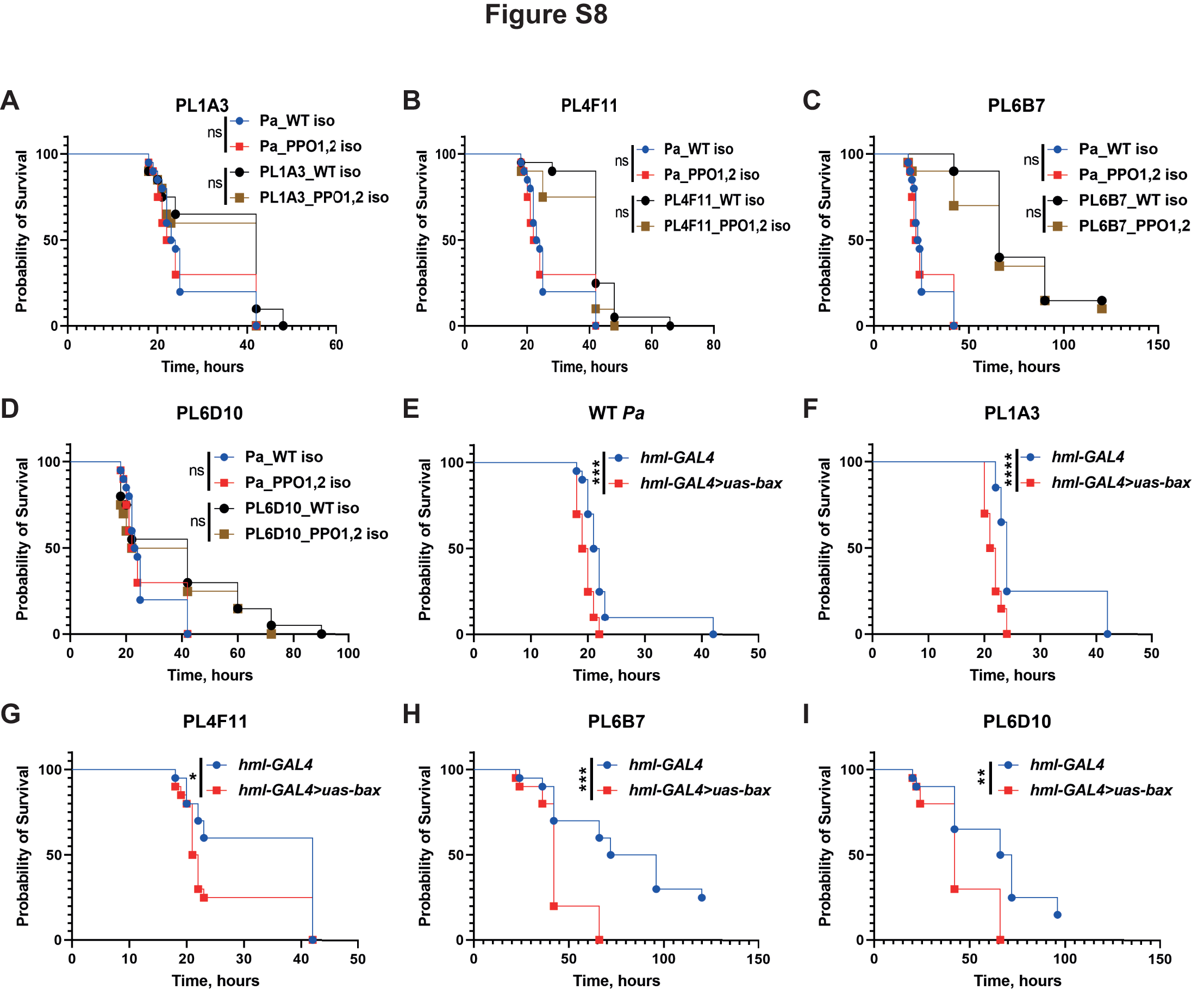

Supplement: S8 Fig — (A-D) ROS sensitive Pa mutants remain less virulent to melanisation-deficient PPO1,2 flies, suggesting that melanisation has no major role in the defense against Pa and is unlikely source of ROS. (E-I) Genetic ablation of hemocytes significantly increases susceptibility to infection with ROS-sensitive Pa mutants. (TIF) [file ppat.1010825.s008.tif]
