# Supplementary material for: Lipopolysaccharide -mediated resistance to host antimicrobial peptides and hemocyte-derived reactive-oxygen species are the major Providencia alcalifaciens virulence factors in Drosophila melanogaster
Source: PLoS Pathog. 2022 Sep 9;18(9):e1010825. doi: 10.1371/journal.ppat.1010825 (PMC9491580; doi:10.1371/journal.ppat.1010825)
Supplement: Attachment — Submitted filename: Point by point responses to the referees comments.docx [file ppat.1010825.s012.docx]

**Point by point responses to the referees comments on Plos Pathogens manuscript PPATHOGENS-D-22-00890** **entitled “Lipopolysaccharide-mediated resistance to host antimicrobial peptides and hemocyte-derived reactive-oxygen species are the major Providencia alcalifaciens virulence factors in Drosophila melanogaster“.**

First of all, we would like to thank the reviewers for their fair comments, helpful suggestions, and constructive criticisms. We believe that we have addressed their concerns and that the new results have improved the manuscript.

We highlighted in yellow all the new figures that we have added while text modifications made during revision are shown in blue in the manuscript.

Reviewer's Responses to Questions

**Part I - Summary**

Reviewer #1: In their publication, Shaka and collaborators showed by “forward” and “reverse” approaches that LPS-mediated resistance to host antimicrobial peptides and hemocyte-derived reactive-oxygen species are the major Providencia alcalifaciens virulence factors in Drosophila melanogaster. The paper is very-well written and the hypothesis are well-driven. However, I have major points that are concerning for this paper to be published in PLoS Pathogens.

Reviewer #2: In this manuscript, Shaka and colleagues describe virulence mechanisms of Providencia alcalifaciens in a Drosophila melanogaster infection model. P. alcalifaciens is an opportunistic pathogen of humans and other Providencia species are frequently used in the study of Drosophila immunity. The authors use genetic manipulation of both bacteria and host to demonstrate that P. alcalifaciens uses LPS modification to evade AMP-mediated host defenses and ROS detoxification as an anti-immune mechanism, arguing that these mechanisms of negating host defenses constitute major virulence mechanisms of P. alcalifaciens in D. melanogaster. The authors also find that host sequestration of iron also plays a major role in controlling P. alcalifaciens infection, and I wonder why they do not emphasize that more in the in Abstract. The work presented is thorough and makes elegant use of reciprocal genetic manipulation in host and pathogen. The findings are novel and interesting, and the article is very well written.

Reviewer #3: This MS by Shaka et al. exploits the power of Drosophila as a model to study host-pathogen interactions. The authors study Providencia alcalifaciens virulence and the host immune responses essential to control this pathogen. The strengths of the work are the unbiased and exhaustive functional approaches deployed by the authors and the robustness, clarity and span of the phenotypes observed. I only have very minor comments listed below. This work is of the highest standard in the host-pathogen field and illustrate another time the power of the fly model to probe in unbiased ways the key mechanisms shaping host-pathogen interactions outcomes both in the host and in the pathogen.

R. We are grateful to reviewers for recognizing the quality of our work and the power of our experimental model.

**Part II – Major Issues: Key Experiments Required for Acceptance**

Reviewer #1: The main message is the demonstration that LPS and ROS resistance are crucial for virulence of Providencia alcalifaciens. However, LPS and ROS resistance are always associated with the pathogenicity of Gram negative pathogens and this has been highlighted by the paper of Yuan et al., (<https://doi.org/10.3389/fmicb.2020.572642>) in their comparative genomic analysis.

R. We feel like the first paragraph of Discussion section addresses the concerns of this reviewer regarding the significance of our findings:

“The aim of our study was to dissect the host-pathogen interactions between Providencia and D. melanogaster. To achieve this aim, we utilized various genetic approaches that enabled us to determine the contributions of both pathogen and host to the outcome of the infection. First, we characterized the responses of the fruit fly to Pa infection and, using mutant analysis, identified the Imd pathway and iron sequestration as prominent defense mechanisms against Pa. Second, we performed an unbiased forward genetics screen using a transposon mutant library that we generated for this purpose and identified Pa virulence factors necessary to infect the fly. Third, we used mutants of the major immune pathways in Drosophila and infected them with attenuated Pa mutants to identify pathogen virulence factors that allow the bacteria to respond to specific immune defenses and evade immune clearance. Thereby, we dissected both sides of host-pathogen relationship in a Drosophila-Providencia model and provided the first insights into the molecular mechanisms of Pa virulence.”

To go further and publish in PLoS Pathogens, this story needs to be decipher with at least the demonstration with isogenic mutants of Pa corresponding to their transposon and the respective complementation.

R. We understand the importance of clean mutants and tried hard to generate such mutants in Pa using several vectors. So far, our attempts were not successful. Considering the difficulties that we encountered and the lack of published mutagenesis protocols for Pa, we believe that efficient mutagenesis protocol has to be established before attempting such large scale mutant generation as the reviewer proposes. We hope that the reviewer will agree that the amount of work and resources needed to accomplish such a task for all 23 mutants is not justified relative to new insights that can be gained from such an approach and impact on major conclusions that we have already drawn.

It was already known that Polymyxin B targets LPS (e.g. <https://doi.org/10.1039/D1FD00036E>). Then I don’t get the point of the authors to use Polymyxin B as a AMP to show that LPS is linked to AMP resistance?

R. The aim of this experiment was to show in vitro that LPS mutants are sensitive to AMPs. We did try 3 AMPs from flies (S2 Table) but they did not show activity. Therefore, we used polymyxin B (cationic AMP, widely used for such purposes). See for example these papers that followed the same strategy: [PMC2928790](http://www.ncbi.nlm.nih.gov/pmc/articles/pmc2928790/), [PMC7775485](http://www.ncbi.nlm.nih.gov/pmc/articles/pmc7775485/) .

Does Drosophila produce an AMP that shows a similar mode of action than Polymyxin B?

R. Very likely, yes. Because LPS-mutants are as virulent as wild-type Pa in flies lacking AMPs but are attenuated in wild-type flies. Therefore, LPS-mutants, which are sensitive to pol B, are also sensitive to one or several AMPs produced by wild-type flies. This means that some Drosophila AMPs, Drosocin for example, act similarly to polymyxin B.

Reviewer #2: The authors use RNAseq to document activation of host immune responses to infection. The RNAseq data are not described in much detail and therefore do not give much insight beyond what was shown with the qPCR data. However, the presented analysis is sufficient for supporting the authors’ main claim that the host immune system is activated by P. alcalifaciens infection, and deposit of the RNAseq data into public databases will allow subsequent researchers to make more thorough use of them.

R. We added one more figure (Fig 2C) to the revised manuscript to extend the description a bit.
However, we decided to focus the description of RNAseq results around the most important point that we wanted to make and let others explore any other specific aspects in our dataset.

The authors use synthetic or purified AMPs to test whether the Pa mutants they recover from their screen are more susceptible to AMPs but find that the AMPs do not restrict bacterial growth in in vitro assays. This was disappointing, I am sure, and I can think of a few possible reasons for it. One is that the tested peptides may indeed have no effect on this bacterium, following the logic of papers like that of Hanson et al (cited ref 57) showing that AMP effects on particular bacteria can be quite specific (indeed, the authors eventually suggest that Drosocin is the primary AMP controlling Pa infection, and Drosocin is not tested in the in vitro assays). Additionally, other authors have shown that in vitro effects of AMPs can be different than in vivo effects of mutants or knockdowns for those same AMPs (see papers from Jens Rolff’s group), suggesting that physiological context or interaction among peptides is important. Furthermore, several investigators from multiple teams informally discuss having had quite a bit of trouble getting in vitro AMP assays to work effectively for a variety of technical reasons (AMPs adhering to plastic assay wells instead of distributing in solution, effects of pH and other chemistry, etc) but this is unfortunately not citable because it is largely unpublished. The authors of the present study do find that the LPS-mutant bacteria are susceptible to polymyxin B, although this AMP is not produced by Drosophila. They also find that Relish mutant hosts are highly susceptible to infection by the mutant Pa, which implies that they are rescued from AMP control in the mutants, but of course Relish also regulates other defense genes in addition to AMPs. The cleanest test of the hypothesis that the LPS-defective mutants are susceptible to AMPs comes from the observation that the Hanson strain of D. melanogaster lacking 10 AMPs and the strain lacking Drosocin specifically are more susceptible to infection by the mutant bacteria. Since these are the definitive experiments, my suggestion would be to move that experiment to the top of the paragraph that begins on Line 298. The authors could subsequently describe the other experiments, including a direct comparison of death rates between Relish and AMP-deletion mutants to demonstrate the AMPs are the major Relish-regulated mechanism of defense. The manuscript could also include some discussion of the limitations of the in vitro assays. These comments are all for the authors to consider and incorporate at their discretion – I am not trying to force a change to the content of the manuscript here, only sharing my perspective on these experiments and my understanding of how they work (or fail to work) in other labs.

R. We agree with these arguments and added a paragraph to the discussion which addresses potential problems with AMPs in vitro assays. We decided not to change the current presentation flow since presenting in vivo data first would make the in vitro results of little value.

The authors do an overexpression rescue with Drosocin and conclude that the rescue means that Drosocin is “particularly important against Pa infection”. A valuable control here would be to test overexpression of other AMPs and show that they do not rescue. It is possible that overexpression of any AMP above physiological levels will control Pa infection and that this effect is not specific to Drosocin. On the other hand, finding that the effect is specific to Drosocin would be a very compelling result that would complement the observation of susceptibility of the specific Drosocin deficiency.

R. As the reviewer suggested, we tested overexpression of the other AMPs and found that, for example, Drosomycin cannot rescue *Relish* mutant. This result is shown in a new Fig S6P.

The experiments documenting the contributions of ROS to host defense and the susceptibility of the Pa mutants to host ROS are nicely performed and I am convinced by them. I appreciate the multiple approaches, including biochemical interventions, the Duox and SOD mutations, and the use of the flies with ablated hemocytes. On line 393, the authors should explicitly state that “hemoless” refers to those flies or eliminate the word from the manuscript since it is not a standard genetic notation.

R. Thank you! We replaced “hemoless” with “hemocyte-deficient”.

The authors do a forward genetic screen in P. alcalifaciens to identify mutants that show reduced infectivity and virulence. As a control, the document that the mutants do now show compromised growth in LB. This is an essential control, especially because mutants with altered LPS may well have altered rates of cell division, but the authors should also note bacterial growth inside of a fly might be quite different that growth in LB (different nutrient availability, etc). The ideal experiment would be to measure rate of proliferation inside of a fly, similarly to what was done in cited ref 59 in the study, particularly because small variations in growth rate could have large effects or host survival time. I understand that it would be difficult to separate growth defects from variation in sensitivity to host immune defense in such an experiment, but perhaps it could be approximated by using Drosophila mutants. I recognize that this would be a large effort and I would not recommend doing it for all mutants recovered from the screen. However, at the authors’ discretion, it may be worth doing so for one or two key bacterial mutants representing the processes that they believe are driving resistance to host defenses.

R. As the reviewer suggested, we measured the proliferation rate of wild-type Pa and of two Pa mutants inside of wild-type and Relish mutant flies (new Fig S4). Consistent with ref 59, we found that wild-type Pa proliferated in both wild-type and Relish mutant flies indistinguishably, as if infection is not controlled at all. However, both Pa mutants that we tested were able to grow only in Relish mutant and their load remained stable in wild-type flies. This result supports the conclusion that Pa mutants do not generally exhibit impaired growth within the fly, but their growth is restricted by the Imd pathway. Interestingly, wild-type flies do not completely clear Pa mutants and carry stable pathogen load. So, it seems that even AMP-sensitive mutants have a way to chronically infect the immunocompetent host.

The forward screen did not reveal any effectors such as bacterial toxins, which the authors found surprising (line 503). However, it seems possible that no such mutants were recovered from the screen because toxins are likely to be redundant so individual mutants may not have much phenotype. Nevertheless, one might have expected mutations in secretion systems to have emerged from the screen, particularly since P. alcalifaciens should have an intact Type III Secretion System. The absence of T3SS mutants in the screen could imply that translocation of effectors is not a mechanism that Pa uses to infect Drosophila.

R. Thank you for the interpretation! We have added this to the discussion.

There is no change for the authors to make in the present study, but the use of the w[1118] strain of Drosophila melanogaster as a nominal wildtype is disappointing. They have done so because many of the host mutants they use in this study are generated in the w[1118] background, so from a genetic perspective, the experiments are done correctly. However, the white mutation is known to be massively pleiotropic and the w[1118] strain is susceptible to infection relative to other Drosophila genotypes. Especially because the mutations studied in this work on not on the X chromosome, the better way to have done this experiment would have been to cross the white-mutant X out of the stocks and conduct the study in a wildtype background. I emphasize that I am not asking the authors to do this now or to repeat this study, and this comment is in no way intended to be a block on publication of the current work. I am only asking the authors to consider avoiding this weak genotype in future studies.

R. We are grateful for the suggestion and aware of potential issues with the w[1118] background. Therefore, we performed survivals with additional wild-type stocks in Figure 1. Since w[1118] did not show any extreme behaviour, we decided to use it, also to avoid issues with the wild-type controls for our immune mutants.

Reviewer #3: NA

**Part III – Minor Issues: Editorial and Data Presentation Modifications**

Reviewer #1: 1- line 168 “at OD10 they were all killed within 20h” – This OD should appear in the mat and met

R. We specified ODs in the Materials and Methods.

2- line 214 to 217 “Our transcriptomic analysis shows that Drosophila…implying that the high virulence of Pa is not due to the inability of the host to recognize or respond to infection”. Did the author compare with a less “highly virulent” strain?

R. We suspect that the reviewer wants to make the point that a less virulent strain might be better recognized and trigger stronger immune response compared to P. alcalifaciens implying that although P. alcalifaciens triggers immune response it might be not as strong or efficient as in case of a less virulent strain. We have not tested such a possibility. However, this study <https://www.sciencedirect.com/science/article/pii/S1286457911000554?via%3Dihub>

showed that P. alcalifaciens induces even stronger IMD pathway activation compared to the mildly-virulent P. rettgeri. To avoid potential confusion, we rephrased the sentence.

3- Figure 5: O-antigen. Which band is it?

R. We have marked the bands in Figure 5.

4- line 354 “prefed”: nothing is written about that in the mat and met. How do the authors perform this experiment?

R. It is described in “Bacterial strain, survival, and bacterial load” section.

5- line 558-559 “surviving flies were counted at regular intervals for 5 days after infection”: the authors should specify more precisely what are “regular intervals”.

R. Specified.

Reviewer #2: The introduction and discussion are very nicely written. They are clear, comprehensive, scholarly, and easy to read.

R. Thank you!

Line 131 states that no strains of other Providencia species are intracellularly invasive. This should probably say “no currently described strains” since it is possible that some unstudied strains possess this ability.

R. Modified.

Line 173, replace “fast” with “quickly”

R. Replaced.

Although I understand the argument the authors are making, there is some logical inconsistency in the paragraph that begins on line 219. They show that infected flies have activated Toll and JAK-Stat pathways, but that mutants of these pathways are not more susceptible to infection. Thus, the infection activates the pathway but activation does not contribute to defense. However, Imd pathway mutants are more susceptible to infection so the authors conclude “this is consistent with the upregulation of imd-responsive genes by Pa”. It is internally inconsistent to conclude that the activation does not necessarily imply effect with Toll and JAK-Stat, but that activation is consistent with an expectation of effect in Imd. The more natural conclusion from these results is that transcriptional activation is not, by itself, a good predictor of effect. This would be a valuable point to emphasize for the entire community: the genes that are transcriptionally responsive to a treatment are not necessarily the genes that are driving the physiological response to the treatment. An experimental manipulation is required to demonstrate effect, inference from transcript levels is not sufficient.

R. We share the reviewer’s view, but we were not clear in expressing it. We deleted “this is consistent with the upregulation of imd-responsive genes by Pa” and added a sentence which hopefully better interprets the results. We agree that experimental manipulation is required to demonstrate the effect and our survivals with fly mutants clearly support this as only some of the transcriptionally upregulated genes/pathways appeared to be relevant.

The references will need to be formatted prior to publication, including correction of a number of the URLs in the reference list.

R. Formatted.

Reviewer #3: Minor comments:

-The authors start by using males and then mention females at some point, I guess clarifying from the beginning if the traits under study are sex-biased or not is important. Then the authors may choose to focus on only one sex.

R. We agree that sexual dimorphism is an important factor to consider. We have not investigated sex differences in response to P. alcalifaciens, therefore we cannot say anything about sex-biases. Most of the reported experiments were performed in male flies, while females were used for RNAseq and qPCR experiments only, merely due to the higher RNA yield. We stated this clearly in materials and methods.

-In Fig3 it would have been nice to test spz mutants for survival assays and I am wondering if the pathogen load is impacted in Toll or Jak/stat pathway mutants.

R. We performed these experiments and they are shown in a new Fig. S1.

-Also in some key fly loss of function conditions where survival to Pa is impacted (Fig6D-E, Fig8G) maybe the authors shall use diluted concentration of the pathogens or its mutants in order to better see the phenotypical windows.

R. We followed the suggestion and infected the flies with a lower dose. Overall, this only delayed the death of the flies, however didn’t improve the resolution (differences between the treatments). Therefore, we left the original graphs in the manuscript.

-On a more general and semantic note I am wondering is mechanisms allowing better tolerance/resistance of the pathogen to host AMP and ROS activities can be qualified as bona fide bacterial virulence factors…? These mechanisms clearly do contribute to the virulence of the pathogen but I feel they may not be qualified as « virulence factors » per se. Maybe the other shall expend on this semantic issue in their discussion.

R. With this comment the reviewer raises a long-debated question: what is a virulence factor? Indeed, historically, a virulence factor was recognized as a component of a pathogen that causes damage to host cells and/or tissues (e.g. toxins). However, with revised concepts of pathogenicity and virulence (<https://pubmed.ncbi.nlm.nih.gov/11443560/>, <https://pubmed.ncbi.nlm.nih.gov/9184008/> ), the categorization of microbial factors that contribute to virulence has become muddied. Currently, a molecule or structure that enables the pathogen to evade or modulate host defense systems to its replicative advantage is also considered a virulence factor. This is also reflected in the most complete definition of virulence that we found (<https://www.ebi.ac.uk/chebi/searchId.do?chebiId=72316> ): “Virulence factors are cellular structures, molecules and regulatory systems that enable microbial pathogens to achieve colonisation of a niche in the host, **inhibit or evade the host's immune response**, enter and exit cells, or obtain nutrition from the host.”

Since resistance to AMPs and ROS help P. alcalifaciens to evade Drosophila immune defenses, these mechanisms fulfil the definition of virulence factors.
